# The Phase Equilibria of Natural Gas Hydrate in the Presence of 1,3-Dimethylcyclohexane and Octyl-β-D-glucopyranoside

**DOI:** 10.3390/molecules29153604

**Published:** 2024-07-30

**Authors:** Qiang Fu, Mingqiang Chen, Weixin Pang, Zengqi Liu, Zhen Xu, Xin Lei

**Affiliations:** 1 State Key Laboratory of Offshore Natural Gas Hydrate, China National Offshore Oil Corporation, Beijing 100028, China; fuqiang8@cnooc.com.cn (Q.F.); pangwx@cnooc.com.cn (W.P.); leixin4@cnooc.com.cn (X.L.); 2 Research Institute of China National Offshore Oil Cooperation, China National Offshore Oil Corporation, Beijing 100028, China; 3 State Key Laboratory of Heavy Oil Processing, China University of Petroleum-Beijing at Karamay, Karamay 834000, China

**Keywords:** gas hydrate, phase equilibrium, methane, surfactant, thermodynamics

## Abstract

The thermodynamic effect of octyl-β-D-glucopyranoside (OGP) on the formation of methane-1,3-dimethylcyclohexane (DMCH) hydrate was studied in this work. The thermodynamic equilibrium hydrate formation pressures between 275.15 K and 283.15 K were measured by the isothermal pressure search method. Different OGP aqueous solutions (0, 0.1, and 1 wt%) were used in this work. The experimental results show that OGP had no obvious thermodynamic inhibition on methane-DMCH hydrate formation when its concentration was low (0.1 wt%), whereas it had an inhibition on methane-DMCH hydrate formation when its concentration was high (1 wt%). The phase equilibrium hydrate formation pressure of the methane-DMCH-OGP system is about 0.1 MPa higher than that of the methane-DMCH system. The dissociation enthalpies of methane hydrate in different solutions remained uniform, which indicates that OGP was not involved in methane-DMCH hydrate formation. This phenomenon is explained from the perspective of the molecular structure of OGP. As a renewable and biological nonionic surfactant, the concentration of OGP in the liquid phase is low, so OGP can be added to the methane-DMCH system without significant thermodynamic inhibition.

## 1. Introduction

Gas hydrates are non-stoichiometric crystals formed by water and guest molecules. The guest molecules are gas and liquid molecules, which are trapped in cages of water molecules, like methane [1], carbon dioxide [2], tetrahydrofuran (THF) [3], cyclopentane (CP) [4], methylcyclohexane (MCH) [5], and 1,3-dimethylcyclohexane (DMCH) [6]. Generally, there are three clathrate structures which are structure I (sI), structure II (sII), and structure H (sH). Due to the cage-like structure of gas hydrates, they can be applied in many fields (like gas storage, carbon dioxide capture and storage, gas separation, seawater desalination, etc.). In addition, natural gas hydrates (NGHs) are alternative energy resources with huge potential, and studies have shown that NGHs have more than double the organic carbon reserves of other fossil fuels [7].

All of those utilizations of NGHs depend on the study of phase equilibrium conditions (pressure or temperature) of NGHs [8]. When the current pressure is lower than the phase equilibrium pressure (*P_eq_*), or the current temperature is higher than the phase equilibrium temperature (*T_eq_*), the NGH dissociates; otherwise, the NGH forms. Milder *P_eq_* or *T_eq_* means lower pressure or high temperature, which is near atmospheric pressure and room temperature. Milder *P_eq_* or *T_eq_* results in low energy consumption to achieve hydrate formation. Therefore, studies of *P_eq_* (or *T_eq_*) are the key factor for controlling the formation or dissolution of NGHs.

**Figure 1:**
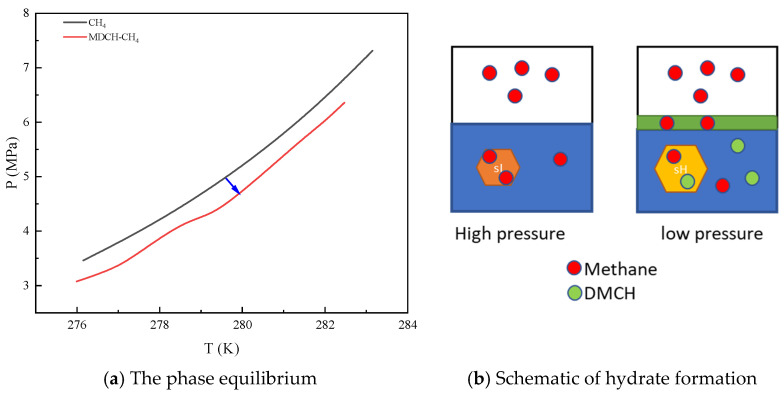
The effect of thermodynamic promoters on *P_eq_*.

The liquid molecules (like tetrahydrofuran, cyclopentane, methylcyclohexane, etc.) that can form sII or sH hydrates are thermodynamic promoters. At the same temperature, the *P_eq_* of sH and sII hydrate with methane are lower than the *P_eq_* of sI with methane only, as shown in Figure 1. Methylcyclohexane, 1,2-dimethylcyclohexane and DMCH cannot form hydrates only by themselves [6], whereas both methylcyclohexane and DMCH can form sH hydrate with methane [6,9], hydrogen [10], or xenon [11]. Hence, the DMCH is used as a kind of thermodynamic promoter in the literature [6].

During the process of hydrate formation, the rate of formation is crucial to application. n-octyl-β-D-glucopyranoside (OGP) is a kinetic promoter, which can accelerate the hydrate formation rate in the literature [12]. The foaming ability of OGP is significantly weaker than the most widely used surfactant as a kinetic promoter, sodium dodecyl sulfate (SDS) [13]. Foams reduce the mass transfer of the gas components between the gas and hydrate phase, and a stronger foaming ability means a lower conversion rate of water into hydrate. OGP also increases the conversion rate of water into hydrate for structure I hydrate [13]. Therefore, OGP is used in this work as a kinetic promoter.

Since the thermodynamic and kinetic effects on hydrate formation are both crucial to the selected promoter, combinations of thermodynamic and kinetic promoters were proposed. There are some combinations for sII hydrates, such as CP +SDS [4], THF + SDS [14,15], THF + nonanoic acid [16], and so on. All of these combinations accelerate the hydrate formation rate and decrease *Peq*. In this work, a combination of promoters (OGP + DMCH) was proposed for the first time for application with low formation pressure and a fast formation rate. Different from the above combinations, OGP + DMCH is a combination of an oleophilic organic chemical and nonionic surfactant, which can reflect the role of nonionic surfactants in sH hydrate formation. The combination of promoters can be used in hydrate-based gas storage of natural gas and the separation of raw natural gas. The low formation pressure and fast formation rate help reduce energy consumption in applications.

## 2. Results

### 2.1. Thermodynamic Consistency

**Figure 2:**
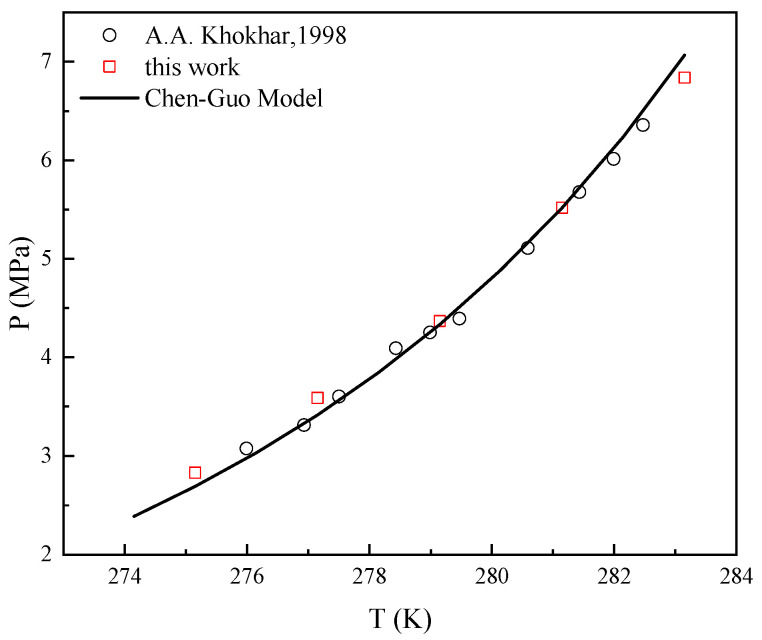
Equilibrium conditions with experimental and literature data and Chen-Guo hydrate model for methane-DMCH-water system. A.A. Khokhar, 1998, is the literature source [6].

The accuracy of the experimental methods and devices used in this study was quantitatively investigated through a comparison with literature data [6]. The van der Waals-Plateeuw model [17,18] and the Chen-Guo model [19] are the most used theoretical models for the *P_eq_* of sH hydrate formation pressure. The two models have similar accuracy for the *P_eq_* of sH hydrate formation pressure, but the Chen-Guo model is simpler to calculate [19]. As shown in Figure 2, the experimental data with the methane-DMCH-water system is in good agreement with the literature data [6] and the Chen-Guo hydrate model. The average relative deviation (ARD) of the Chen-Guo model is 2.8% for the methane-DMCH-water system [19]. The values of *P_eq_* data were all within the prediction interval of the Chen-Guo model.

To explore the thermodynamic effects of DMCH-methane hydrates in the presence of OGP on ΔH_diss,_ we carried out calculations based on data of *P_eq_* using the Clausius-Clapeyron equation [20,21]:

(1) $$\frac{\mathrm{dln}P_{eq}}{d\frac{1}{T_{eq}}}=\frac{\Delta H_{\mathrm{diss}}}{zR}$$

where *T_eq_* and *P_eq_* are the equilibrium temperature and pressure of the DMCH-methane hydrates measured in this work; z is the compressibility factor of the methane gas under the equilibrium conditions (*T_eq_* and *P_eq_*), which is calculated by the Patel-Teja equation of state [22]; *R* is the universal gas constant.

A method of verifying the thermodynamic consistency of the experimental data was used in this work [23,24]. This method consists of three assessments. The first assessment used in the work is based on the Clausius-Clapeyron equation.

The assessment verifies the statistical goodness (1 − R^2^) of linear regression for the following equation:

(2) $$lnP_{eq}=A/T_{eq}+B$$

where A and B are the constants in linear regression. The values of 1 − R^2^ within 2% indicate good reliability in a narrow temperature range of about 10–20 K.

**Figure 3:**
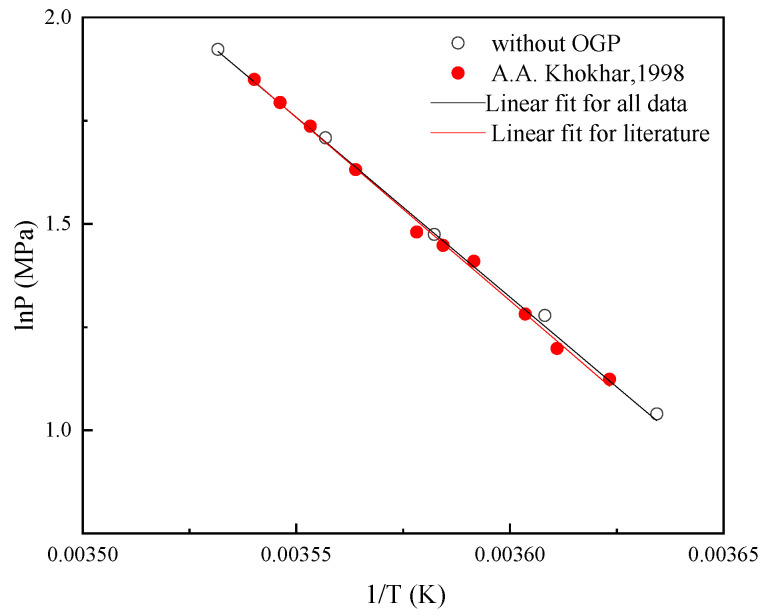
Results of thermodynamic consistency. (A.A. Khokhar, 1998) is the literature source [6].

To effectively assess gas hydrate phase equilibrium data from different sources, thermodynamic consistency was verified. Figure 3 shows the assessment results of thermodynamic consistency. The statistical goodness (1 − R^2^) in the first assessment for the methane-DMCH-water system is 0.37% for literature data [6] and 0.36% for all data (experimental and literature data). Both of the statistical goodness values are less than 2%, which is reported in the literature [23]. The second assessment of thermodynamic consistency, based on the differences in water activity, is then applied to different systems. The experimental and literature data are the same system with the same water activity. The second assessment is not applied to this system.

### 2.2. Thermodynamic Effect on P_eq_

**Figure 4:**
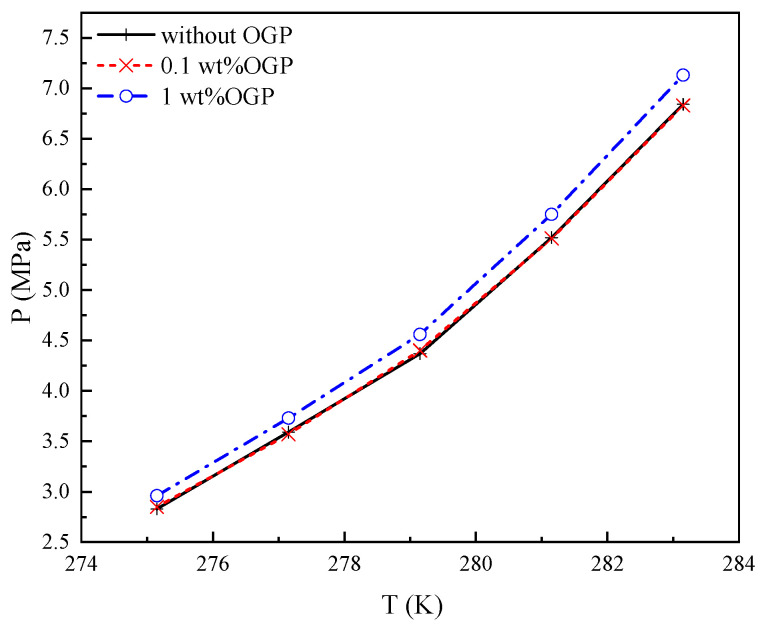
Equilibrium conditions of methane-DMCH-OGP-water system with different concentrations of OGP (0.1 and 1 wt%).

The value of *P_eq_* for the methane-DMCH-OGP-water system was measured with different concentrations of OGP (0.1 and 1 wt%) in the temperature range of 275.15 K to 283.15 K. The results are displayed in Figure 4.

As illustrated in Figure 4, *P_eq_* for the methane-DMCH-OGP-water system increased with the increase in temperature, which is the same trend as the methane-DMCH-water system. *P_eq_* for the methane-DMCH-OGP-water system decreased with the increase in the concentrations of OGP, which is the same as other surfactant additives for sII hydrates [25]. Differences in *P_eq_* between systems in the absence and presence of 0.1 wt% OGP (±0.02 MPa) highlight the uncertainties of the experimental result (±0.01 MPa). However, *P_eq_* for the system in the presence of 1 wt% OGP is higher than in the absence of OGP. The difference in *P_eq_* is more than 0.1 MPa.

To confirm the thermodynamic effect on *P_eq_*, the Clausius-Clapeyron equation (Equation (1)) is used in this work for calculating the ΔH_diss_ of sH hydrate. The results of ΔH_diss_ are listed in Table 1. The results show that ΔH_diss_ of the methane-DMCH-water system (71.0, 71.3 kJ mol^−1^) is close to that of the methane-DMCH-OGP-water system (71.1 kJ mol^−1^). The difference in ΔH_diss_ is for 0.1 wt% and 1 wt% OGP, respectively.

## 3. Discussion

### 3.1. Thermodynamic Effect on P_eq_

As a kind of emulsifier, the low concentration of OGP in the solution can improve the effect on the mixing of oil (DMCH) and water phases [26]. The mechanism will be discussed in Section 3.2. As illustrated in Figure 4, when the OGP concentration is 0.1 wt%, the emulsifier effect on *P_eq_* is the main effect, and the thermodynamic effect is not significant. When the OGP concentration is 1 wt%, the thermodynamic effect is the main effect. OGP does not participate in hydrate formation, and its hydroxyl groups bind to water by hydrogen bonds. Therefore, the hydroxyl effect has a negative thermodynamic effect on *P_eq_*.

Generally, kinetic promoters cannot participate in hydrate formation and do not affect hydrate crystal structure. As listed in Table 1, ΔH_diss_ remains constant, which confirms that OGP has the same effect on *P_eq_* as other kinetic promoters. This indicates that OGP is only present in the liquid (water or oil) phase, not the hydrate phase. During the process of hydrate formation, the moles of water and DMCH decrease in the liquid phase and increase in the hydrate phase, while the OGP remains in the liquid phase. The increase in OGP concentration in the liquid phase increases the negative effect on *Peq*_._ This indicates that the amount of the kinetic promoters used for hydrate formation should be strictly limited to very small amounts like 0.1 wt%.

### 3.2. Mechanism of OGP in Hydrate Formation

**Figure 5:**
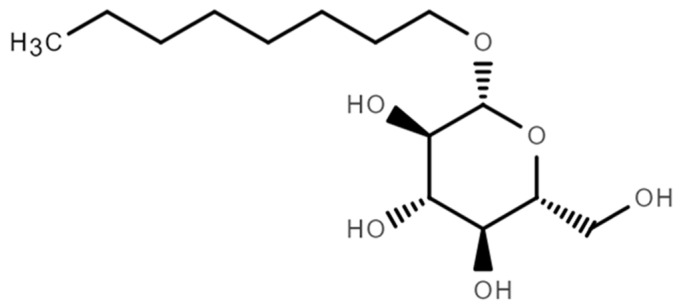
The structural formula of OGP.

OGP is a kind of surfactant, also called an amphiphile [27]. The structural formula of OGP is shown in Figure 5. As shown in Figure 5, one OGP contains four hydrophilic hydroxyl groups and one oleophilic n-octyl group.

**Figure 6:**
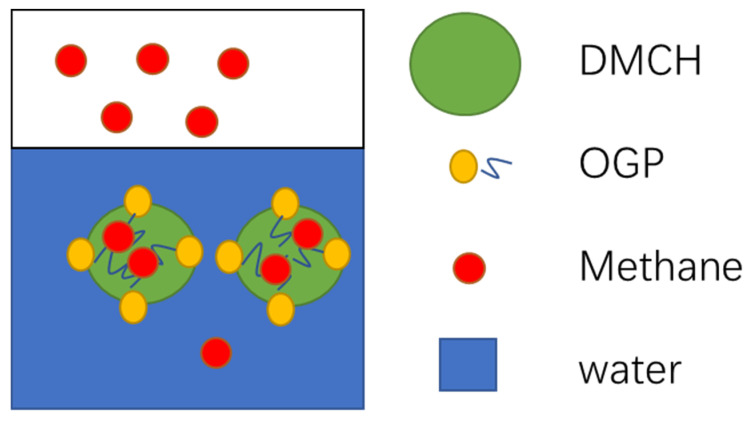
Schematic of OGP as a kind of emulsifier.

In the mixture of DMCH and water, the DMCH is the oleophilic phase and the water is the hydrophilic phase, as shown in Figure 1. The hydroxyl groups of OGP are soluble in the hydrophilic phase because of forming hydrogen bonds with water, and the n-octyl groups are soluble in the oleophilic phase [28] as shown in Figure 6. OGP is an emulsifier, as shown in Section 3.1. The emulsion or microemulsion of DMCH-OGP-water has a better mixing effect than the phase-splitting mixture of DMCH-water because there is a larger contact between oil and water. Similarly, OGP plays a similar role in the dissolution of methane. Methane is soluble in the oleophilic phase but insoluble in the hydrophilic phase. OGP can help methane become soluble in the hydrophilic phase. This is the mechanism of OGP promoting hydrate formation as a kinetic promoter via the increase in the oil-water or gas-liquid contact area and solubility.

However, the forming of hydrogen bonds with water does not always have a positive effect on hydrate formation. The hydrogen bonds between the water and OGP experience close bonding. The hydrates are formed by hydrogen bonds between water and water. The hydroxyl groups in OGP and water are competitive, the water-water bond has a positive effect on hydrate formation [29], and the OGP-water bond has a negative effect on hydrate formation. That means that more OGP molecules prevents further hydrate formation. This is the mechanism of OGP in terms of its thermodynamic inhibition effect on hydrate formation.

In conclusion, OGP has two effects on hydrate formation: the kinetic promotion effect in the 0.1 wt% OGP system and the thermodynamic inhibition effect in the 1 wt% OGP system. Generally, the amount of surfactant in the hydrate formation system is less than 1wt%, so it always shows the kinetic promotion effect on hydrate formation. However, it cannot be neglected that the concentration of OGP can increase during the hydrate formation process. Because the higher concentration of OGP has a thermodynamic inhibition effect on hydrate formation, the thermodynamic equilibrium will shift to less hydrate formation.

## 4. Materials and Methods

### 4.1. Materials and Apparatus

The experimental gas is pure methane (purity ≥ 99.99 mol%), which was provided by Beijing Yongsheng Gas Technology Industry Company (Beijing, China). MDCH (purity ≥ 97%) was provided by Aladdin Biochemical Technology Company (Shanghai, China). OGP (purity ≥ 96%) was provided by Shanghai Acmec Biochemical Company (Shanghai, China). Deionized water (18 × 10^6^ Ω·cm) and MDCH were weighed by an electronic balance (±0.1 mg).

**Figure 7:**
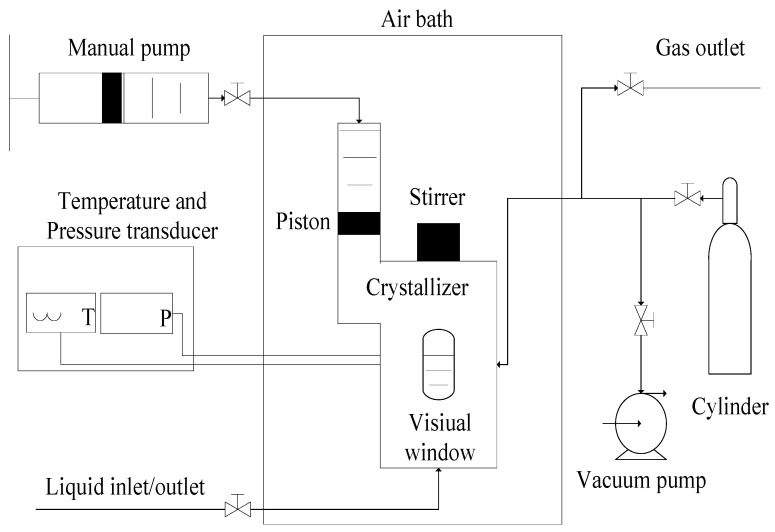
Schematic diagram of experimental apparatus for the measurements of the equilibrium hydrate formation conditions.

Figure 7 shows the experimental apparatus which was used in this work. The crystallizer volume is adjusted by using the manual pump with scale division lines (uncertainty of ±0.05 mL), and its maximum value is 465.0 mL. The uncertainties of the measured pressure and measured temperature are ±0.005 MPa and ±0.05 K, respectively.

### 4.2. Experimental Method

The concentration of DMCH was set at 17 wt% because the concentration of DMCH in the solution approaches the mole rate of water-DMCH in sH hydrates.

The concentration of OGP was set at 0, 0.1, and 1 wt%, respectively. The critical micelle concentration (CMC) exists for any surfactant. If the concentration of the surfactant is more than the CMC, the properties of the solution, like interfacial tension, will be changed. The CMC of OGP in different oils and water is within the range of 0.1 and 1 wt% [30].

The experimental temperature is within the range of 275.15–283.15 K, which is the operating temperature of hydrate-based gas storage and gas separation. When the temperature is less than 273.15 K, the liquid water will form ice and reduce the amount of hydrate; when the temperature is more than 283.15 K, the *P*_eq_ will be more than 7 MPa, which is higher than the pressure of natural gas wells.

The *P*_eq_ of methane-liquid (DMCH-water or DMCH-OGP-water) systems was measured by the pressure search method [23]. The experimental procedure was as follows:

1. The crystallizer was washed with deionized water and then washed with the target liquid at least three times.
2. The crystallizer was purged at least three times using methane to completely remove air from the system.
3. The 40 mL target liquid was added to the crystallizer.
4. The crystallizer volume was set at its maximum value (465.0 mL) when the gas was introduced into the crystallizer.
5. The air bath was switched on to maintain the experimental temperature in the crystallizer.
6. When the temperature in the crystallizer was stabilized at the experimental temperature for at least 15 min, the pressure in the crystallizer was increased gradually by adjusting the manual pump until a small trace of hydrate was observed.
7. The pressure was set at the estimated value.
8. If the trace of hydrate disappeared in 4 h, this indicated that the estimated value was lower than *P_eq_*. The estimated pressure value was increased, and the experiment was repeated from Step 5.
9. If the amount of hydrate increased, indicating that the estimated value was higher than the equilibrium pressure at the experimental temperature, the pressure was decreased to completely dissociate the hydrate. The estimated value of the pressure was decreased, and the experiment was repeated from Step 6.
10. If the trace of hydrate crystals lasted for more than 4 h (indicating that the estimated value was equal to the equilibrium pressure at the experimental temperature), the data for equilibrium hydrate formation were recorded.
11. P_eq_ under each experimental condition was measured at least three times. The uncertainty of *P*_eq_ is ±0.01 MPa.

### 4.3. Thermodynamic Framework

In this work, we used the Chen-Guo thermodynamic model for sH hydrate [19] which used the Patel-Teja equation of state (EoS) [22] and PT flash to calculate the fugacity of methane and DMCH. In this work, the cubic-plus-association (CPA) EoS [31] is used to calculate the fugacity of methane and DMCH instead of the Patel-Teja EoS, because the CPA used association parameters for water is better for gas-water phase equilibria.

Phase equilibrium conditions are determined by chemical potential equilibria. When the chemical potential change (Δ*μ*) is 0, the pressure is equal to *P*_eq_. The Δ*µ* can be calculated by the following equation [19,23]:

(3) $$\Delta \mu =\mathrm{RT}\left[\lambda_{3}ln\frac{f_{MDCH}^{H}}{f_{MDCH}}+\lambda_{1}ln\left(1-\theta_{1}\right)+\lambda_{2}ln\left(1-\theta_{2}\right)\right]$$

where $\theta_{1}$ and $\theta_{2}$ are the occupation fraction of the smallest and middle linked cages in hydrates filled by methane, respectively. $f_{MDCH}$ and $f_{MDCH}^{H}$ are the fugacity of the MDCH in the liquid phase and the basic hydrate under the experimental condition, respectively. $f_{MDCH}$ is calculated by CPA EOS. $\lambda_{1}$ and $\lambda_{2}$ are the ratio of the smallest and middle linked-cage numbers to the water-molecule number, respectively. $\lambda_{3}$ is the ratio of the basic-cage number to the water-molecule number. $\lambda_{1}$, $\lambda_{2}$ and $\lambda_{3}$ are determined by the hydrate structure: $\lambda_{1}$ is 3/34, $\lambda_{2}$ is 2/34, and $\lambda_{3}$ is 1/34 in sH hydrates [19].

Based on the Langmuir adsorption theory, *θ* can be expressed as follows [32]:

(4) $$\theta_{i}=\frac{f_{g}C_{i}}{1+f_{g}C_{i}}$$

where *i* stands for the gas in the linked cages; 1 and 2 stand for smallest and middle linked cages, respectively. $f_{g}$ is the fugacity of methane calculated by CPA EOS. $C_{i}$ is the Langmuir constant of methane correlated as an Antoine-type equation:

(5) $$C_{i}=X^{\prime }\exp\left(\frac{Y^{\prime }}{T-Z^{\prime }}\right)$$

where $X^{\prime }$, $Y^{\prime }$, and $Z^{\prime }$ are the Antoine parameters from the model [17], as shown in Table 2.

Based on the Chen-Guo model, $f_{DMCH}^{H}$ is calculated as follows [17,32]:

(6) $$f_{DMCH}^{H}=f_{T}^{H}\left(T\right)\cdot \exp\left(\frac{\beta P}{T}\right){\cdot \alpha }_{w}^{-\frac{1}{\lambda_{3}}}$$

where *β* is the parameter of hydrate structure, which is 22.288 K/MPa for sH hydrates [17]. $\alpha_{w}$ is the water activity determined by pressure-temperature flash calculation based on the CPA EoS [17]. $f_{T}^{H}\left(T\right)$ is a function of temperature in the Chen-Guo model. $f_{T}^{H}\left(T\right)$ is recommended to be written as follows:

(7) $$f_{T}^{H}\left(T\right)=A^{\prime }\exp\left(\frac{B^{\prime }}{T-C^{\prime }}\right)$$

where $A^{\prime }$ is 2.692 × 10^76^ MPa, $B^{\prime }$ is −92,097.15 K and $C^{\prime }$ is −248.22 K.

CPA EOS can be described as two terms: a cubic term ($p^{c}$) and an association term ($p^{a}$) [33]. The cubic term can be any cubic equation of state, like SRK EoS [33], PR EoS [34], and other Modified EoS [35]. In order to be consistent with the Chen-Guo model, the Patel-Teja EoS [17] was used as a cubic term, which is written as follows:

(8) $$\begin{array}{c} p=p^{c}+p^{a}=\frac{RT}{\nu -b}-\frac{a}{\nu \left(\nu +b\right)+c\left(v-b\right)}\\ \;\;\;\;\;\;\;\;\;\;\;\;\;\;\;\;\;\;\;\;\;\;\;\;\;\;\;\;\;\;\;\;\;\;\;\;\;\;\;\;\;\;\;\;\;\;\;\;\;\;\;\;\;\;\;-\frac{1}{2}\frac{RT}{\nu }\left[1+\rho \frac{\partial \ln g}{\partial \rho }\right]\sum_{i}\;x_{i}\sum_{A_{i}}\;\left(1-X_{i}^{A}\right)\; \end{array}$$

where a, b, and c are the parameters of Patel-Teja EoS provided in the literature [23]. And g and ρ can be written as follows:

(9) $$\Delta_{A_{i}B_{j}}=g\left(\rho \right)v_{A_{i}B_{j}}\left[\exp\left(\frac{\varepsilon_{A_{i}B_{j}}}{k_{B}T}\right)-1\right]$$

(10) $$g\left(\rho \right)={\left(1-\frac{1.9b}{4v}\right)}^{-1}$$

The parameters of association term $v_{A_{i}B_{j}}$ and $\varepsilon_{A_{i}B_{j}}$ are provided in the literature [33,34,35].

## 5. Conclusions

In this study, *P*_eq_ of methane-DMCH hydrate was compared in different OGP concentrations, 0, 0.1, and 1 wt%, and in the temperature range of 275.15–283.15 K. The effects of OGP were described as two effects on hydrate formation. The conclusions are as follows:

(1) The kinetic promotion effects of OGP on *P*_eq_ were described as an emulsifying effect, and that effect was the main effect in the 0.1 wt% OGP system, which had no significant thermodynamic effect on *P*_eq_ because of the low concentration.
(2) The thermodynamic effect on *P*_eq_ was the main effect in the 1 wt% OGP system, which showed a negative effect because of the hydroxyl groups of OGP bonding to water by hydrogen bonds.
(3) The Clausius-Clapeyron equation was used to explore the thermodynamic effects of DMCH-methane hydrates in the presence of OGP on ΔH_diss_. The results show that ΔH_diss_ of the methane-DMCH-water system was close to that of the methane-DMCH-OGP-water system (around 71.0 kJ mol^−1^) and it remains constant, which confirms that OGP had the same effect on *P_eq_* as other kinetic promoters, and OGP was only presented in the liquid (water or oil) phase and was not in the hydrate phase.
(4) Furthermore, these effects can guide the number of kinetic promoters used for hydrate formation for gas storage, gas separation, and other natural gas hydrate research.

## Figures and Tables

**Table 1:** Calculated dissociation enthalpies with different OGP concentrations.

OGP Concentrations (wt%)	Calculated Dissociation Enthalpies (kJ∙mol^−1^)
0	71.1
0.1	71.3
1	71.0

**Table 2:** The parameters used for the modeling.

	$X^{\prime }$(Pa)	$Y^{\prime }$(K)	$Z^{\prime }$(K)
$C_{1}$	2.3048 × 10^−11^	2752.29	23.01
$C_{2}$	1.433 × 10^−10^	2625.04	19.93

## Data Availability

Data are contained within the article.
